# *Methylocystis hydrogenophila* sp. nov.—A Type IIa Methanotrophic Bacterium Able to Utilize Hydrogen as an Alternative Energy Source

**DOI:** 10.3390/microorganisms13102309

**Published:** 2025-10-05

**Authors:** Kangli Guo, Thomas Heimerl, Anna Hakobyan, Dongfei Han, Werner Liesack

**Affiliations:** 1 Department of Functional and Evolutionary Ecology, University of Vienna, Djerassiplatz 1, 1030 Vienna, Austria; 2 Center for Synthetic Microbiology (SYNMIKRO), Philipps University of Marburg, 35043 Marburg, Germany; heimerl@staff.uni-marburg.de; 3 Molecular Biology of the Rhizosphere, Institute for Crop Science and Resource Conservation (INRES), University of Bonn, 53115 Bonn, Germany; anna.hakobyan@uni-bonn.de; 4 School of Environmental Science and Engineering, Suzhou University of Science and Technology, Suzhou 215009, China; dongfeihan@usts.edu.cn; 5 Independent Researcher, 35037 Marburg, Germany

**Keywords:** *Methylocystis*, digital DNA–DNA hybridization, phylogenomics, chemotaxonomy, hydrogenases, asparagine

## Abstract

A novel species of the genus *Methylocystis* is proposed based on polyphasic evidence from strain SC2^T^, isolated from the heavily polluted Saale River near Wichmar, Germany. Digital DNA–DNA hybridization and phylogenomic analyses demonstrate that strain SC2^T^ represents a distinct species within the genus, clearly separated from its closest relatives, namely *Methylocystis suflitae* NLS-7^T^, *Methylocystis rosea* SV97^T^, *Methylocystis silviterrae* FS^T^, and *Methylocystis hirsuta* CSC1^T^. As is typical of the family *Methylocystaceae*, cells possess intracytoplasmic membranes arranged parallel to the cytoplasmic membrane, and the dominant fatty acids are C18:1ω8c and C18:1ω7c. The strain grows aerobically on methane as the primary carbon and energy source and expresses both low- and high-affinity particulate methane monooxygenase (pMMO), but lacks the soluble form. The species epithet reflects the strain’s ability to utilize hydrogen as an alternative energy source. A further feature is its use of asparagine as an osmoprotectant, enhancing salt tolerance. Genomic analysis reveals complete pathways for nitrogen fixation, denitrification, and hydrogen oxidation. These genetic and physiological characteristics support the designation of a novel species, for which the name *Methylocystis hydrogenophila* sp. nov. is proposed. The type strain is SC2^T^ (=DSM 114506 = NCIMB 15437).

## 1. Introduction

Proteobacterial methane-oxidizing bacteria, or methanotrophs, are capable of utilizing methane as their sole source of carbon and energy. These organisms include species belonging to the classes Alphaproteobacteria (Type IIa and IIb) and Gammaproteobacteria (Type Ia, Ib, and Ic) [1]. To date, five genera of cultivated alphaproteobacterial methanotrophs have been described: *Methylocystis* and *Methylosinus* (family *Methylocystaceae*, Type IIa), as well as *Methylocapsa*, *Methylocella*, and *Methyloferula* (family *Beijerinckiaceae*, Type IIb) [1].

Members of the genus *Methylocystis* were the first methanotrophic Alphaproteobacteria to be isolated and described, by Whittenbury in 1970 [2]. These bacteria are characterized by the presence of intracytoplasmic membranes (ICMs) arranged parallel to the cytoplasmic membrane, a predominance of C_18_ fatty acids, and the utilization of the serine pathway for carbon assimilation. *Methylocystis* species exhibit broad ecological versatility, reflecting their adaptability to a wide range of environmental conditions. They play important roles in methane cycling within methanogenic habitats such as peat bogs, rice paddies, freshwater sediments, landfills, and permafrost, where they act as natural biofilters. In addition, they contribute to atmospheric methane oxidation in upland soils [3,4,5,6,7].

Although methane is their principal source of carbon and energy, some *Methylocystis* species have been shown to grow slowly on multi-carbon substrates such as ethanol and acetate [8]. They are also capable of utilizing various nitrogen sources [9] and can accumulate poly-β-hydroxybutyrate under nutrient-limited conditions [10].

To date, the genus *Methylocystis* comprises ten species with validly published names (https://tygs.dsmz.de/, accessed on 8 July 2025):

*Methylocystis suflitae* NLS-7^T^ [11], *Methylocystis hirsuta* CSC1^T^ [12], *Methylocystis silviterrae* FS^T^ [13], *Methylocystis rosea* SV97^T^ [14], *Methylocystis borbori* 9N^T^ [15], *Methylocystis iwanois* SS37A-Re^T^ [16], *Methylocystis parvus* OBBP^T^ [17], *Methylocystis echinoides* IMET 10491^T^ [18], *Methylocystis heyeri* H2^T^ [19], and *Methylocystis bryophila* H2s^T^ [20]. In addition to these validly described taxa, the genus *Methylocystis* includes numerous taxonomically uncharacterized strains, underscoring its widespread, cosmopolitan distribution. *Methylocystis* is recognized as one of the most abundant and functionally active groups of methanotrophs across diverse terrestrial ecosystems [1].

The initial and most crucial step in methane oxidation is catalyzed by the enzyme methane monooxygenase (MMO) [21]. There are two distinct forms of MMO: the particulate, membrane-bound methane monooxygenase (pMMO), which is universally present in members of the genus *Methylocystis*, and the soluble, cytoplasmic methane monooxygenase (sMMO), which has been identified in only a subset of methanotrophic species [22,23,24]. The expression of sMMO is restricted to low-copper conditions, and it exhibits a broader substrate specificity than pMMO, rendering it particularly valuable for biotransformation and bioremediation applications. In contrast, pMMO is copper-dependent and has a relatively narrow substrate range [22]. The pMMO enzyme complex comprises three polypeptides, including PmoC, PmoA, and PmoB, and is encoded by the *pmoCAB* operon. Compared to sMMO, the pMMO is the environmentally relevant MMO.

Strain SC2^T^ was the first member of the genus *Methylocystis* demonstrated to encode two pMMO isozymes; a low-affinity and high-affinity form, which together enable the strain to oxidize methane across a broad concentration range. The low-affinity enzyme, pMMO1, is encoded by two *pmoCAB1* gene clusters, while the high-affinity enzyme, pMMO2, is encoded by a single *pmoCAB2* gene cluster. The latter facilitates methane oxidation at low atmospheric concentrations (~1.9 ppm), with an apparent Michaelis-Menten constant [*K*_m(app)_] of 0.11 μM. This value aligns with *K*_m(app)_ estimates reported for atmospheric methane oxidation in soil environments [25,26,27,28].

Over the past 15 years, a combination of physiological assays and multi-omics approaches, including genomics, transcriptomics, proteomics, metabolomics, and ^13^C-tracing, has been applied to comprehensively characterize the phenotypic and genomic features of strain SC2^T^. In this study, we provide a detailed description of its phenotypic, genotypic, and metabolic traits in comparison with its closest phylogenetic relatives. Based on these analyses, we propose the designation of strain SC2^T^ as a novel species within the genus *Methylocystis*, for which the name *Methylocystis hydrogenophila* sp. nov. is proposed. The species epithet reflects the key physiological characteristic of strain SC2^T^ to utilize hydrogen as an alternative energy source under conditions where both methane and oxygen are limited [29].

## 2. Materials and Methods

### 2.1. Isolation and Cultivation of Strain SC2^T^

Strain SC2^T^ was isolated from the highly polluted Saale River near Wichmar, Germany [25,30]. A serial dilution series was prepared using tubes containing 5 mL of basal salts medium A supplemented with NH_4_Cl. The tubes were incubated at 30 °C in sealed glass desiccators containing a gas mixture of 20% (*v*/*v*) CH_4_, 5% CO_2_, and 75% air. Samples from the terminal positive dilution step were streaked onto plates containing medium A and incubated under the same gas atmosphere. Individual colonies were selected under a stereo microscope and repeatedly restreaked onto fresh plates to obtain a pure culture, designated SC2^T^.

Strain SC2^T^ was maintained in mineral salt medium (MS) as previously described, with the following composition per liter: Na_2_HPO_4_·2H_2_O, 0.5 g; KH_2_PO_4_, 0.22 g. One milliliter (0.1%, *v*/*v*) of a trace element solution was added, containing (g L^−1^): EDTA, 5.0; FeSO_4_·7H_2_O, 2.0; ZnSO_4_·7H_2_O, 0.1; MnCl_2_·4H_2_O, 0.03; H_3_BO_3_, 0.05; CoCl_2_·6H_2_O, 0.2; CuCl_2_, 0.06; NiCl_2_·6H_2_O, 0.02; Na_2_MoO_4_, 0.03. Stock solutions of MgSO_4_ and CaCl_2_ were prepared with 2.0 g MgSO_4_·7H_2_O and 0.4 g CaCl_2_·2H_2_O per 20 mL, respectively; 1 mL of each stock solution was added per liter of medium. Additionally, 1.0 g of nitrate was added as the nitrogen source, yielding the nitrogen mineral salts (NMS) medium [30].

For growth in liquid culture, 120-mL serum bottles were used, with a liquid-to-headspace ratio of 1:2. The headspace was filled with a filter-sterilized gas mixture of 80% air and 20% CH_4_ (*v*/*v*). Cultures were incubated at 25 °C on a rotary shaker at 150 rpm.

The purity of the culture was routinely verified by (i) phase-contrast microscopy and (ii) whole-cell fluorescence in situ hybridization using the general bacterial probe EUB338 (green), the species-specific probe Mcyst-1256 (red), and counterstaining with DAPI (blue) [25].

### 2.2. 16S rRNA Gene and Phylogeny

The 16S rRNA gene sequences of thirteen validly published species within the family *Methylocystaceae*, seven species from the family *Beijerinckiaceae* (type II methanotrophs), and one representative strain from the family *Methylococcaceae* (type I methanotrophs) were retrieved from GenBank for phylogenetic analysis. Sequence alignment was performed using the MUSCLE algorithm. Phylogenetic trees were constructed using both the Neighbor-Joining (NJ) and Maximum Likelihood (ML) methods implemented in MEGA version 12 [31,32,33]. Tree topology confidence was assessed using 1000 bootstrap replicates [34].

### 2.3. Genomic Sequencing, Analysis and Annotation

To determine the taxonomic placement and characterize the genomic features of strain SC2^T^, total genomic DNA was extracted and subjected to whole-genome shotgun sequencing using the 454 GS-FLX Titanium platform [35]. Approximately 0.5 million reads were obtained, with an average read length of 380 bp. Genome assembly was carried out using the MIRA assembler [36], and contigs were further extended and curated via primer walking and manual editing in Consed [37]. Coding sequences (CDSs) were predicted using GLIMMER version 2.1 [38]. Circular plasmid maps were generated using DNAPlotter [39], and genome annotation of both the chromosome and plasmids was conducted as previously described [35].

A phylogenomic analysis was performed using a concatenated alignment of 120 single-copy phylogenetic marker genes identified with GTDB-Tk version 2.4.0 [40]. The phylogenetic tree was inferred using IQ-TREE version 2.4.0 [41] and visualized with MEGA12. Pairwise average nucleotide identity (ANI) values between strain SC2^T^ and closely related type strains were calculated using FastANI [42]. Average amino acid identity (AAI) was computed using CompareM (https://github.com/dparks1134/CompareM accessed on 8 July 2025). Digital DNA–DNA hybridization (dDDH) values were obtained through the Type Strain Genome Server (TYGS) [43].

### 2.4. Physiological and Chemotaxonomic Analysis

Physiological tests assessing the effects of temperature, pH, and NaCl concentration on the growth of strain SC2^T^ were conducted using nitrate mineral salts (NMS) medium supplemented with 20% (*v*/*v*) methane in the headspace as the sole energy and carbon source. Growth was monitored spectrophotometrically at 600 nm (BioPhotometer RS232C; Eppendorf AG, Hamburg, Germany) until the stationary phase. Temperature-dependent growth was evaluated across a range of 4 to 40 °C (specifically at 4, 10, 15, 20, 25, 28, 30, 37, and 40 °C). pH-dependent growth was assessed over a range of pH 3.0 to 8.0, with 0.5-unit increments, in liquid NMS medium; pH adjustments were made according to the method of Tikhonova et al. [13]. The effect of salinity on growth was determined using NaCl concentrations ranging from 0 to 1.5% (*w*/*v*).

The substrate utilization profile of strain SC2^T^ was investigated by testing various carbon sources. Methanol utilization was evaluated in NMS liquid medium, with methanol concentrations ranging from 0.01 to 2% (*v*/*v*); methane was omitted from the headspace in these experiments. Nitrogen source utilization was assessed by substituting KNO_3_ in the NMS medium with ammonium (NH_4_^+^) at concentrations ranging from 1 to 100 mM. Nitrogen fixation capability was tested using nitrogen-free mineral salts medium (NFMS) [35].

Coccoid to rod-shaped morphology of strain SC2^T^ was examined using a Zeiss Axiophot light microscope (Zeiss, Jena, Germany). Cells in the mid-exponential growth phase were harvested for fatty acid analysis. Cellular fatty acids were converted into fatty acid methyl esters (FAMEs) via saponification, methylation, and extraction, following minor modifications of the method described by Sasser [44]. The FAMEs were analyzed by gas chromatography with flame ionization detection, using the Sherlock Microbial Identification System (MIS; MIDI, Microbial ID, Newark, DE, USA). Peak integration and quantification of fatty acids were carried out using the MIS Standard Software. Summed features were further resolved, and fatty acid identities were confirmed by gas chromatography–mass spectrometry (GC-MS) based on retention time locking and mass spectral data. The fatty acid analyses were performed by the Identification Service of the Leibniz Institute DSMZ—German Collection of Microorganisms and Cell Cultures GmbH (Braunschweig, Germany), following the protocols detailed at: https://www.dsmz.de/services/microorganisms/biochemical-analysis/cellular-fatty-acids accessed on 8 July 2025.

### 2.5. Transmission Electron Microscopy

Concentrated cell suspensions were high-pressure frozen using an HPF Compact 02 system (Wohlwend, Sennwald, Switzerland) and subsequently freeze-substituted in an AFS2 unit (Leica, Wetzlar, Germany). The substitution medium consisted of acetone supplemented with 0.25% osmium tetroxide, 0.2% uranyl acetate, and 5% double-distilled water (ddH_2_O), following the protocol: −90 °C for 20 h; warmed to −60 °C over 1 h; held at −60 °C for 8 h; raised to −30 °C over 1 h; maintained at −30 °C for 8 h; and finally increased to 0 °C over 1 h. At 0 °C, samples were washed three times with acetone before being incubated for 2 h at room temperature in a 1:1 mixture of Epon 812 substitute resin (Fluka, Buchs, Switzerland) and acetone. This 1:1 mixture was then replaced with pure resin for overnight infiltration. Following a second exchange with fresh Epon resin, samples were polymerized at 60 °C for 48 h.

Polymerized blocks were trimmed with razor blades and sectioned into 50 nm ultrathin slices using an ultramicrotome (UC7, Leica, Wetzlar, Germany) equipped with a diamond knife (Diatome, Biel, Switzerland). Sections were mounted onto 100-mesh copper grids coated with pioloform. Post-staining was performed with 2% uranyl acetate for 20–30 min, followed by 0.5% lead citrate for 1–2 min. Transmission electron microscopy (TEM) analysis was conducted at 120 kV using a JEM-2100 transmission electron microscope (JEOL, Tokyo, Japan) equipped with a 2 k × 2 k F214 fast-scan CCD camera (TVIPS, Gauting, Germany).

## 3. Results and Discussion

### 3.1. Sequence Identity and Phylogenetic Analysis

**Figure 1:**
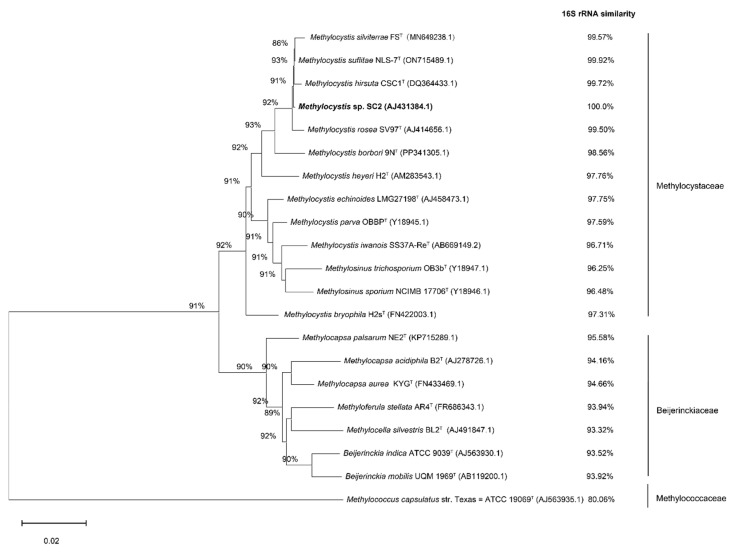
Phylogenetic tree based on 16S rRNA gene sequences, showing the relationship of *Methylocystis* sp. strain SC2^T^ to other *Methylocystis* strains and representatives of related genera. The NJ tree was constructed using MEGA 12. Bootstrap values are based on 100 replications. The 16S rRNA sequence similarity values are shown in comparison to that of strain SC2^T^. Genbank accession numbers are shown in parentheses.

Phylogenetic analysis based on the 1495 bp 16S rRNA gene sequence of strain SC2^T^ revealed that it clusters with the validly described species *Methylocystis suflitae* NLS-7^T^, *Methylocystis rosea* SV97^T^, *Methylocystis silviterrae* FS^T^, and *Methylocystis hirsuta* CSC1^T^ with a bootstrap support value of 92% (Figure 1). The 16S rRNA gene sequence similarity between strain SC2^T^ and these species exceeds 99%; however, none of them shares 100% sequence identity with SC2^T^. Although these values are above the 99.0% similarity threshold often used to delineate bacterial species [45], 16S rRNA gene sequences alone frequently lack sufficient resolution for accurate species-level identification [46].

**Figure 2:**
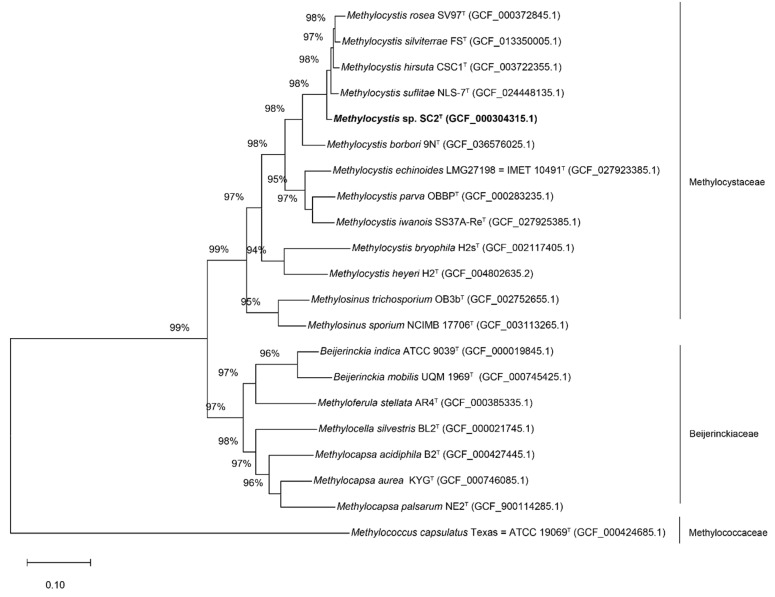
ML phylogenomic tree derived from 120 concatenated single-copy marker proteins showing the position of strain SC2^T^ in relation to the type strains of taxonomically characterized species of the families *Methylocystaceae* and *Beijerinckiaceae*. The phylogenetic analysis was visualized using MEGA12. Bar, 0.10 amino acid substitutions per site. The tree was rooted using *Methylococcus capsulatus* Texas^T^ as the outgroup. GenBank accession numbers are given in parentheses.

To further assess the taxonomic position of strain SC2^T^, genome-based classification was performed using GTDB-Tk in comparison with its closest *Methylocystis* relatives. The genome of strain SC2^T^ comprises three replicons with a total size of 4,146,594 bp, including a circular chromosome (3,773,444 bp) and two plasmids, designated pBSC2-1 (229,614 bp) and pBSC2-2 (143,536 bp), with average G+C contents of 63%, 61%, and 60%, respectively [9]. A phylogenomic tree based on 120 concatenated single-copy marker genes placed strain SC2^T^ within a cluster containing *M. suflitae*, *M. hirsuta*, *M. silviterrae*, and *M. rosea*, corroborating the results of the 16S rRNA gene-based phylogeny (Figure 1 and Figure 2).

Average nucleotide identity (ANI) values between strain SC2^T^ and its closest relatives were 92.55% with *M. suflitae* NLS-7^T^, 92.79% with *M. hirsuta* CSC1^T^, 92.90% with *M. silviterrae* FS^T^, and 91.71% with *M. rosea* SV97^T^ (Table 1). Similarly, the average amino acid identity (AAI) values were 93.36%, 93.40%, 93.44%, and 92.62%, respectively. These values fall below the widely accepted species demarcation threshold of 95% for both ANI and AAI [47], indicating that strain SC2^T^ represents a distinct species within the genus *Methylocystis*.

This conclusion is further supported by dDDH analysis, performed using the Type Strain Genome Server (TYGS; https://tygs.dsmz.de/, accessed on 8 July 2025), which compared the genome of strain SC2^T^ against those of ten validly described *Methylocystis* species. The highest dDDH values observed were 64.5% and 63.7% with *M. suflitae* NLS-7^T^ and *M. silviterrae* FS^T^, respectively (Table 2). These values are well below the 70% threshold commonly used to define bacterial species [48].

Collectively, the phylogenetic, genomic, and dDDH analyses provide robust evidence that strain SC2^T^ represents a novel species within the genus *Methylocystis*, closely related to *M. suflitae*, *M. hirsuta*, *M. silviterrae*, and *M. rosea* (Figure 1 and Figure 2; Table 1 and Table 2).

### 3.2. Cellular and Chemotaxonomic Analysis

For comparative purposes, the cellular characteristics of strain SC2^T^ were analyzed alongside those of the four phylogenetically closest related species: *Methylocystis suflitae* NLS-7^T^, *Methylocystis rosea* SV97^T^, *Methylocystis silviterrae* FS^T^, and *Methylocystis hirsuta* CSC1^T^ (Table 2).

**Figure 3:**
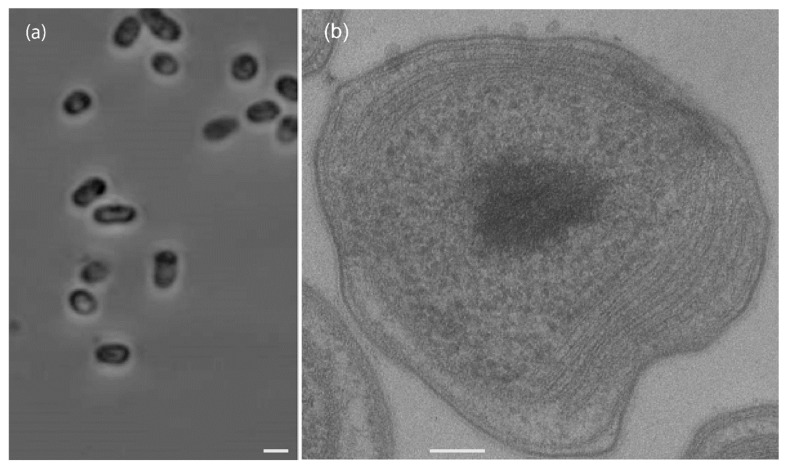
Phase-contrast micrograph of SC2^T^ cells grown in standard NMS medium under methane for 3 days. Bar, 1 µm (**a**). Electron micrograph of the ultrathin section of a single SC2^T^ cell. Bar, 100 nm (**b**).

Colonies of strain SC2^T^ on solid medium were circular, convex, and exhibited entire margins. They were opaque, white, and displayed iridescence. Cellular morphology ranged from coccoid (0.8–1.0 μm in diameter) to rod-shaped forms (1.2–3.5 × 1.0–1.5 μm). Cells were nonmotile and occurred singly, in pairs, or in irregular aggregates. Some cells contained refractile inclusions (Figure 3a). In older agar cultures, enlarged, irregular lipid cysts were observed [25]. Such phenotypic variation in colony and cell morphology is common within the genus and can be influenced by environmental conditions, including medium composition, temperature, and host interactions [49]. Similar morphological plasticity has been described in species such as *M. suflitae* and *M. silviterrae*, which can alternate between distinct morphotypes [11,13].

Ultrathin sections of SC2^T^ cells harvested during exponential growth on methane and NMS medium revealed a well-developed ICM arranged parallel to the cytoplasmic membrane (Figure 3b). This membrane architecture is characteristic of the genus *Methylocystis* [12,13,14].

More than 98% of total fatty acids identified in strain SC2^T^ were C18:1ω7c (35.1%) and C18:1ω8c (63.8%). This composition closely corresponds in both identity and quantity to that detected in the phylogenetically closest relatives, including *Methylocystis suflitae* NLS-7^T^, *Methylocystis hirsuta* CSC1^T^, *Methylocystis silviterraea* FS^T^, and *Methylocystis rosea* SV97^T^ (Table 3).

### 3.3. Growth on Methane and Other Carbon Compounds

Strain SC2^T^ exhibited robust growth across a wide range of methane concentrations when methane was supplied as the sole carbon and energy source, while no growth was detected when methanol was provided. Slight growth on acetate occurred, with an increase in OD_600_ of only 0.007 after 400 h of incubation [50]. Although most members of the genus *Methylocystis* are obligate methanotrophs, several facultative species capable of utilizing multicarbon substrates, such as acetate and ethanol, have been described. These include the moderately acidophilic strains *Methylocystis bryophila* H2s^T^, *Methylocystis heyeri* H2^T^, and *Methylocystis* sp. strain SB2, as well as the mesophilic species *Methylocystis echinoides* IMET10491^T^. This facultative metabolic capacity may confer a selective advantage in environments where methane availability is variable.

All genes required for a methanotrophic lifestyle were identified in the SC2^T^ genome [9,35]. Strain SC2^T^ encodes both low- and high-affinity pMMO isozymes, allowing oxidation of methane over a wide range of concentrations. However, comparative genomic analysis did not reveal genes encoding sMMO. The second step of methane oxidation is the conversion of methanol to formaldehyde by methanol dehydrogenase (MDH). Two distinct MDH systems are encoded by the SC2^T^ genome. One is the well-studied calcium-dependent MDH-*Mxa* type and the other is the lanthanide-containing MDH-*Xox* type. All the genes involved in the serine cycle, encoding serine-glyoxylate aminotransferase (*Sga*), hydropyruvate reductase (*Hpr*), two subunits of malate thiokinase (*MtkAB*), an acetyl-coenzyme A (acetyl-CoA)-independent phosphoenol pyruvate carboxylase (*Ppc*), and malyl-CoA lyase (*Mcl*), were identified. The genes for the two key enzymes of the ribulose monophosphate (RuMP) pathway, 3-hexulose-6-P synthase and hexulose-P isomerase, were not detected, indicating that strain SC2 does not use the RuMP pathway for formaldehyde assimilation.

### 3.4. Physiological Characterization

Strain SC2^T^ exhibited growth characteristics comparable to those of the type strains of closely related *Methylocystis* species (Table 2). It grew within a temperature range of 4 °C to 37 °C, with optimal growth occurring between 25 °C and 27 °C. Growth was supported across a pH range of 5.5 to 8.0, with an optimum at pH 7.0–7.2. Strain SC2^T^ grew in the presence of up to 0.75% (*w*/*v*) NaCl; however, higher salinity tolerance was observed in the presence of osmoprotectants. Among various osmoprotectants evaluated, asparagine exhibited the most pronounced protective effect, enabling strain SC2^T^ to grow at NaCl concentrations as high as 1.5%. The uptake of asparagine by the cells induced significant remodeling of metabolic pathways in strain SC2 [51].

Strain SC2^T^ was capable of utilizing both nitrate and ammonium as nitrogen sources, exhibiting optimal growth at concentrations ranging from 1 to 10 mM [52]. Additionally, the strain was able to fix atmospheric dinitrogen (N_2_) as the sole nitrogen source. Maximum growth under N_2_-fixing conditions was observed at oxygen concentrations between 5% and 10%. The genome of strain SC2^T^ encodes a diverse array of nitrogen metabolism pathways, including transport and assimilation of nitrate and ammonium, hydroxylamine detoxification, denitrification, and dinitrogen fixation. A complete set of 34 genes involved in N_2_ fixation was identified, along with a full denitrification pathway, which includes the plasmid-encoded *nosRZDFYX* operon.

Polyhydroxybutyrate (PHB) biosynthesis proceeds via acetyl-CoA derived from the serine pathway. PHB-related genes detected in the chromosome include those encoding PHB depolymerases, the polyhydroxyalkanoate synthesis repressor (*phbR*), acetyl-CoA acetyltransferase (*phbA*), acetoacetyl-CoA reductase (*phbB*), and phasin homologs. Under N_2_-fixing conditions, PHB accumulation reached 30% of total dry cell mass (mg/mg total solids) [53].

### 3.5. Hydrogen Utilization Capability

Strain SC2^T^ was the first member of the genus *Methylocystis* shown to utilize hydrogen as an alternative energy source. Hydrogen utilization was assessed under varying methane and oxygen conditions. The addition of 2% H_2_ to the culture headspace nearly doubled the biomass yield of strain SC2^T^ under conditions of methane (6%) and oxygen (3%) limitation [29].

**Figure 4:**
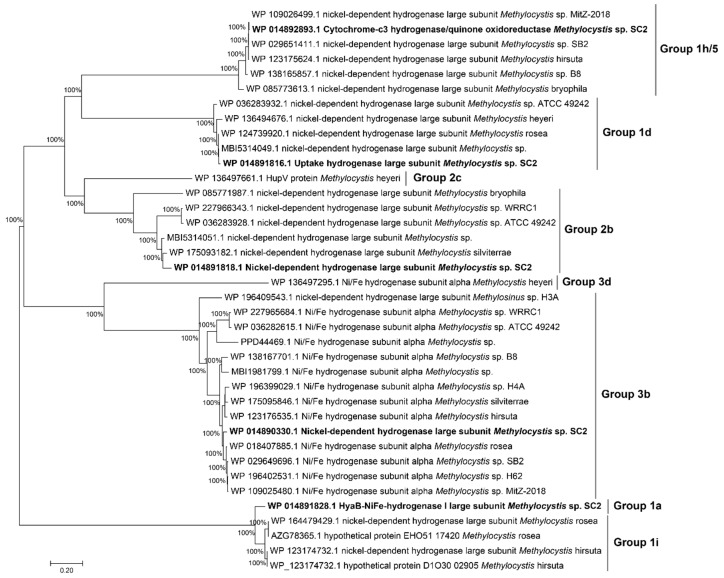
Protein tree of hydrogenases encoded by strain SC2^T^ in comparison to those encoded by other *Methylocystis* spp. The ML tree was constructed with MEGA7. Hydrogenase enzymes were classified into eight groups (1a, 1d, 1h/5, 1i, 2b, 2c, 3b, and 3d). Bar: 0.2 substitutions per amino acid site.

Genomic analysis revealed the presence of genes encoding hydrogenases and associated accessory proteins in strain SC2^T^. Specifically, five distinct hydrogenase types were identified: groups Ia, Id, 2b, 3b, and 1h/5 (Figure 4). Proteomic analysis further demonstrated that hydrogen supplementation markedly increased the abundance of group 1d and regulatory group 2b [NiFe]-hydrogenases. These enzymes likely facilitate the transfer of electrons derived from hydrogen oxidation into the quinone pool under methane- and oxygen-limited conditions. These results suggest a functional link between hydrogen oxidation and energy conservation, implying a metabolic reorganization in response to nutrient limitation.

Notably, while genes encoding hydrogenases and associated accessory proteins were detected across all available *Methylocystis* genomes (Figure 4), experimental evidence for hydrogen utilization as an alternative energy source remains exclusive to strain SC2^T^ [29,54].

## 4. Conclusions

The comprehensive characterization of strain SC2^T^ unequivocally supports its classification as a novel species within the genus *Methylocystis*. Phylogenomic, ANI, AAI, and dDDH analyses consistently demonstrate that SC2^T^ is genetically distinct from its closest relatives, despite exhibiting high 16S rRNA gene sequence similarity. Morphologically and physiologically, SC2^T^ shares the hallmark characteristics of *Methylocystis* species, including intracellular membrane architecture, specific fatty acid profiles, and the ability to grow on methane as the sole carbon and energy source.

However, it also displays several distinctive features, such as enhanced osmotolerance mediated by asparagine, facultative nitrogen fixation with full denitrification capability, and uniquely within the genus the ability to utilize hydrogen as an alternative energy source under methane- and oxygen-limited conditions. These physiological and metabolic traits likely confer ecological advantages in fluctuating environments. Collectively, the integrated genomic, phenotypic, and metabolic data provide a compelling rationale for the recognition of strain SC2^T^ as a new species within the genus *Methylocystis*.

## 5. Description of *Methylocystis hydrogenophila* sp. nov.

*Methylocystis hydrogenophila* (hy.dro.ge.no’phi.la. Gr. n. *hydrogen* hydrogen; Gr. adj. *philos* loving; N.L. fem. adj. *hydrogenophila*, hydrogen-loving, referring to the ability to utilize molecular hydrogen (H_2_) as an additional energy source).

A member of the methanotrophic genus *Methylocystis*, which belongs to the type II methanotrophic Alphaproteobacteria. Cells are Gram-negative, aerobic, and coccoid (0.8–1.0 μm in diameter) to rod-shaped (1.2–3.5 × 1.0–1.5 μm), occurring singly, in pairs, or in irregular aggregates. Cells are non-motile and contain an ICM typical of type II methanotrophs, arranged parallel to the cytoplasmic membrane. Colonies on solid media are circular, convex with entire margins, opaque, white, and display an iridescent sheen.

Growth occurs at temperatures between 4 °C and 37 °C, with an optimum between 25 °C and 27 °C. The pH range for growth is 5.5–8.0, with an optimum at pH 7.0–7.2. Growth is supported in the presence of up to 0.75% (*w*/*v*) NaCl; higher salinity tolerance may be observed in the presence of osmoprotectants such as asparagine. Cells utilize methane as the sole carbon and energy source and express two distinct pMMO isozymes; however, genes encoding sMMO are absent. Cells remain viable for extended periods under atmospheric concentrations of methane. No growth is observed on methanol. Weak growth is supported on acetate. Cells are also capable of utilizing molecular hydrogen (H_2_) as an additional energy source.

Cells are diazotrophic and can utilize dinitrogen (N_2_), nitrate, or ammonium salts as nitrogen sources. Optimal growth is observed with nitrate or ammonium at concentrations of 1–10 mM. Under nutrient-limited conditions, intracellular PHB granules are accumulated. The predominant cellular fatty acids are C18:1ω7c and C18:1ω8c.

The type strain, SC2^T^ (=DSM 114506^T^ = NCIMB 15437^T^), was isolated from the highly polluted River Saale near Wichmar, Germany. The complete genome of strain SC2^T^ comprises a circular chromosome with a DNA G+C content of 63 mol%. Additionally, two plasmids, designated pBSC2-1 and pBSC2-2, possess G+C contents of 61 mol% and 60 mol%, respectively. The genome sequences have been deposited in the EMBL/GenBank/DDBJ databases under the accession numbers HE956757 (chromosome), FO000001 (pBSC2-1), and FO000002 (pBSC2-2). The GenBank accession number for the 16S rRNA gene sequence (1495 bp) of strain SC2^T^ is AJ431384.1.

## Figures and Tables

**Table 1:** ANI, AAI, and dDDH values between strain SC2^T^ and the type strains of validly described *Methylocystis* species.

Organism Name	ANI	AAI	dDDH
*Methylocystis silviterrae* FS^T^ (GCF_013350005.1)	92.90	93.44	63.7
*Methylocystis hirsuta* CSC1^T^ (GCF_003722355.1)	92.79	93.4	62.5
*Methylocystis suflitae* NLS-7^T^ (GCF_024448135.1)	92.55	93.36	64.5
*Methylocystis rosea* SV97^T^ (GCF_000372845.1)	91.71	92.62	60.2
*Methylocystis borbori* 9N^T^ (GCF_036576025.1)	82.50	80.64	27.4
*Methylocystis iwanois* SS37A-Re^T^ (GCF_027925385.1)	80.09	74.9	17.6
*Methylocystis parvus* OBBP^T^ (GCF_000283235.1)	80.01	74.68	17.7
*Methylocystis echinoides* LMG27198 = IMET 10491^T^ (GCF_027923385.1)	80.30	74.2	17.3
*Methylocystis heyeri* H2^T^ (GCF_004802635.2)	78.66	70.04	14.9
*Methylocystis bryophila* H2s^T^ (GCF_002117405.1)	78.40	68.26	14.5

All ANI and AAI values are less than 95% and all dDDH values are less than 70% demonstrating species-level delineation of strain SC2^T^.

**Table 2:** Characteristics that distinguish SC2^T^ from type strains of other closely related species of the genus *Methylocystis*.

Characteristic	1 SC2^T^	2 NLS-7^T^	3 CSC1^T^	4 FS^T^	5 SV97^T^
Isolation source	Polluted river	Landfill cover soil	Ground water	Forest soil	Arctic wetland soil
Cell shape	Rods	Rods, dumbbells	Dumbbell	Small curved coccoids/rods	Rods
Color of colonies	White	Yellow	Cream	Cream	Pink-red
Cell width (μm)	1.0–1.5	0.8	0.3–0.6	0.5–0.7	0.8–1.1
Cell length (μm)	1.2–3.5	1.33	0.7–1.0	1.7–3.4	1.1–2.5
Temperature range for growth (optimum), °C	4–37 (25–27)	10–35 (30)	4–35 (25–30)	4–37 (25–30)	5–37 (27)
pH range for growth (optimum)	5.5–8 (7.0–7.2)	5.5–8.5 (6.5–7.0)	5.8–8.5 (6.5–7.0)	4.5–7.5 (6.0–6.5)	5.0–9.0
NaCl (*w*/*v*, %)	0–0.75%	ND	ND	ND	ND
Growth on acetate (%)	w	−	w	−	−
sMMO	−	−	−	−	−
pMMO2	+	−	−	+	−
DNA G+C content (mol%)	63.4	62.5	62.4	62.6	62

Strains: 1, *Methylocystis hydrogenophila* sp. nov. SC2^T^; 2, *Methylocystis suflitae* NLS-7^T^ [11]; 3, *Methylocystis hirsuta* CSC1^T^ [12]; 4, *Methylocystis silviterrae* FS^T^ [13]; 5, *Methylocystis rosea* SV97^T^ [14]. +, present; −, absent; w, weak growth.

**Table 3:** Cellular fatty acids composition (percentages) of SC2^T^ and related type strains.

Fatty Acid	SC2^T^	NLS-7^T^	CSC1^T^	FS^T^	SV97^T^
16:1ω7c	ND	ND	ND	ND	6.1
16:1ω6c	ND	ND	ND	ND	ND
C16:1ω9c	ND	ND	ND	ND	ND
C16:1ω9t	ND	ND	2.3	ND	ND
C16:0	ND	ND	0.3	ND	ND
C18:1ω8c	63.8	69.2	71.1	74.5	74.8
C18:1ω7c	35.1	28.3	26.1	24.7	23.7
C18:0	ND	ND	0.3	0.8	ND

Strains: 1, Methylocystis hydrogenophila sp. nov. SC2^T^; 2, Methylocystis suflitae NLS-7^T^ [11]; 3, Methylocystis hirsuta CSC1^T^ [12]; 4, Methylocystis silviterrae FS^T^ [13]; 5, Methylocystis rosea SV97^T^ [14]. ND, Not detected.

## Data Availability

The original contributions presented in this study are included in the article. The original sequences presented in the study are openly available at [NCBI] at https://www.ncbi.nlm.nih.gov/nuccore/HE956757 accessed on 8 July 2025; [Uni-ProtKB] at https://www.uniprot.org/taxonomy/187303 accessed on 8 July 2025. Further inquiries can be directed to the corresponding authors.

## Abbreviations

ICMs: intracytoplasmic membranes; pMMO, particulate methane monooxygenase; sMMO; soluble methane monooxygenase; FAMEs, fatty acid methyl esters; NJ, Neighbor-Joining; ML, Maximum Likelihood; dDDH, digital DNA–DNA hybridization; ANI, average nucleotide identity; AAI, average amino acid identity; MDH, methanol dehydrogenase; RuMP, ribulose monophosphate; PHB, polyhydroxybutyrate.
